# Towards comprehensive mental health care: experiences and challenges of psychosocial care in Brazil

**DOI:** 10.1186/s12889-021-11397-1

**Published:** 2021-07-08

**Authors:** Mariá Lanzotti Sampaio, José Patrício Bispo Júnior

**Affiliations:** Multidisciplinary Institute of Health, Federal University of Bahia (UFBA), Vitória da Conquista, Brazil

**Keywords:** Mental health, Deinstitutionalisation, Health policy and systems research, Low- and middle-income countries, Brazil

## Abstract

**Background:**

Recommendations are in place for mental health (MH) care to be developed into a comprehensive, people-centred perspective and organised primarily through community services. In recent decades, Brazil has promoted psychiatric reform aimed at transforming the hospital-centred model into a psychosocial model of MH. However, current political and economic changes threaten this reform. This article analyses the comprehensive MH care offered by a Psychosocial Care Network (*Rede de Atenção Psicossocial* – RAPS) in Brazil.

**Methods:**

The study involved semi-structured in-depth interviews with 33 stakeholders (policymakers, health professionals, and MH service users) and direct observation of MH services members of the RAPS. Data were analysed using framework analysis with the following dimensions: mental health services access, long-term mental health care, comprehensive mental health care, and crisis patient care.

**Results:**

Results indicated progression towards comprehensive MH care provision. We identified MH care provided primarily by community services, featuring an ‘open door’ policy, development of localised actions and a search for autonomy. Deinstitutionalisation principles and the psychosocial model support a comprehensive view of MH by policy makers, MH professionals, and users. However, difficulties in providing comprehensive care remain, with the main challenges being insufficient services offered and difficulties in user access at all levels of care, fragile integration between services, lack of clear definitions of the responsibilities of each service, discontinuity of care, limitations in family support, and fragility in crisis patient care.

**Conclusion:**

We highlight the need to increase funding and services of RAPS, qualification of staff professional, family support, and development of strategies for integrating services. Support and expansion of MH care depend on strengthening the Brazilian health system, which is in danger of being dismantled.

**Supplementary Information:**

The online version contains supplementary material available at 10.1186/s12889-021-11397-1.

## Background

Mental health (MH) care models are widely debated by international organisations, scholars, policy makers, professionals, and service users. Hospital-centred approaches have been strongly questioned because they are exclusionary and restrictive. In low- and middle-income countries (LMICs), the approaches to MH care commonly involve abuse and human rights violations [1]. Since the mid-twentieth century, several countries have initiated psychiatric reform movements to transform mental hospitals and reform their restrictive practices [2].

Currently, international guidelines on MH policies focus on within-community care, articulated in various care domains and supported by a people-centred health paradigm and social recovery strategies [3, 4]. Emphasis has shifted from disease to people and their contexts, with active participation of users, family members, communities, and services [4]*,* as well as focusing on autonomy and social integration. In 2013, the World Health Organisation [5] established the Comprehensive Mental Health Action Plan 2013–2020, in which it urged member countries to strengthen effective MH leadership and governance; provide comprehensive, integrated, and responsive community-based MH and social care services; implement MH promotion and prevention strategies; and support research and scientific evidence for MH.

Although advances in global MH have occurred, there are still weaknesses in the technical-care transformation, which still has limited reach [6]. Many countries still show difficulties in MH service access [7], MH-related human rights violations [6], insufficient social integration [8], care not centred on individuals and their family members [9], and weaknesses in response to crisis patients [10]. Moreover, service provision quality and funding are commonly below those of physical health services [6].

Despite advances, difficulties in properly addressing MH issues are even greater in LMICs. Petersen et al. [11] identified the following main difficulties of integrated and expanded MH care in LMICs: restrictions on financial and human resources, care fragmentation between sectors, and fragile MH assessment indicators. According to those authors, when MH indicators are not collected by national health information systems, there may be difficulties in monitoring quality of care and identifying cases. Wakida et al. [9] estimated that a high proportion (up to 85%) of people with severe mental disorders do not receive adequate and effective treatment in these countries. In Latin America, a high proportion of people with severe mental disorders remain untreated [12, 13]. Kohn et al. [13] revealed a treatment gap of around 74.7% for moderate and severe disorders in Latin American countries.

In Brazil, MH care is integral to the Unified Health System (*Sistema Único de Saúde* – SUS). This is a universal system, financed by general taxes, and based on comprehensive care and equal access [14]. Brazilian Psychiatric Reform (*Reforma Psiquiátrica Brasileira* – RPB) developed in parallel and in line with Brazilian Sanitary Reform [15]. RPB has made progress in shaping a psychosocial and community care model, replacing the previous hospital-centred model [16].

The country’s Mental Health Policy was largely restructured with the promulgation of Law 10,216 of 2001 [17], known as the Brazilian Psychiatric Reform Law. This law repealed the archaic MH legislation [18], prohibited establishment of new beds in psychiatric hospitals [19], and set financing mechanisms for community-based care services [18]. In 2011, the Psychosocial Care Network (*Rede de Atenção Psicossocial* – RAPS) was established, whose objectives were to ensure the articulation and integration of health services at all levels, expand the population’s access to psychosocial care, and qualify care through attendance, continuous monitoring, and emergency care [20].

RAPS consist of several services: primary health care (PHC), including Family Health Support Centres (*Núcleos Ampliados de Saúde da Família* – NASF) that have MH professionals on staff; community MH services (Psychosocial Care Centres – *Centros de Atenção Psicossocial* – CAPS); hospital services, with priority for psychiatric beds in general hospitals and systematic reduction of beds in psychiatric hospitals; and deinstitutionalisation strategies, social support, work, and income [16, 19, 21]. RPB has enabled important advances, such as drastic reduction of beds in psychiatric hospitals from 53,962 in 2001 to 25,998 in 2014; reversal of MH expenditure flow, with community services now receiving more resources than psychiatric hospitals; and considerable expansion of CAPS and therapeutic residential services [22].

Within this context, we emphasise the need for deeper understanding of these ongoing transformations and analysis of the challenges of translating theoretical constructs from the psychosocial model to technical-care practice. Challenges related to chronic underfunding of the health system [23], governance and evaluation problems [24], and human resource development [22] interfere in the practical operationalisation of changes intended to suit this purpose. In this article, we aimed to analyse MH care performance of a psychosocial care network in Brazil in the movement towards comprehensive MH care.

## Methods

### Study design

This is a qualitative case study of the technical assistance aspects of MH care in Brazil. We were interested in investigating key dimensions in the delivery of MH care in services at different levels of care and organised in networks. Our study considered international guidelines [4, 6, 25] that advocate construction of integrated community-based care MH networks, with user centrality, and which are systematically organised, albeit in a flexible way, based on scientific evidence and human rights conventions.

### Study setting

The study was conducted at the RAPS in Vitória da Conquista, state of Bahia, north-eastern Brazil. The municipality is the third largest in the state, with a population of 338,885 inhabitants, a GDP per capita of $8700, and a human development index of 0.678, in the medium human development range [26]. The municipality is the seat of the Health Region of Southwest Bahia and receives patients referred from 82 other small and medium-sized municipalities to deliver secondary health services.

The RAPS consists of services at the three levels of care. In PHC, the municipality has 45 family health teams (*Equipes de Saúde da Família* – eSF), seven Traditional Basic Health Units, five NASF, and one Street Clinic team (*Consultório da Rua*). Specialised outpatient MH care has a CAPS intended for children and adolescents (CAPS-IA), a CAPS for severe and persistent mental disorders (CAPS-II), and another for users of alcohol and other drugs (CAPS-AD). In addition to the three abovementioned CAPS, there are two psychiatric outpatient clinics. For hospital and emergency care, there is a mobile emergency medical rescue service (*Serviço de Atendimento Móvel de Urgência* – *SAMU*), three emergency hospital attendances, and an MH ward in a general hospital [27].

In 2017, the psychiatric hospital, which operated in the municipality for 60 years, was closed and MH beds were opened at the Regional General Hospital. Since then, MH care in the locality has been reorganised, encouraging decentralisation of hospital care to the open and community environment.

### Data sources and participants

Two complementary methods were used to obtain data: semi-structured in-depth interviews and direct observation. This approach allowed comprehensive analysis of MH assistance by integrating the views of the various stakeholders with observations on practices developed in the various RAPS services. MLS conducted in-depth interviews and direct observations. This author is a psychologist with a master’s degree in public health and experience in the field of mental health, hospital psychology, and care for the homeless.

Thirty-three semi-structured in-depth interviews were held with 21 healthcare professionals, seven policy makers, and five MH users. The number of participants per type of service is summarised in Table 1. Policy makers and professionals were selected according to their position at the RAPS. We recruited policy makers who were managers of services and of the general hospital. Professionals were selected who provided direct care to users in the services studied, with the aim of encompassing the various professional categories. For users, inclusion criteria were being over 18 years old, being lucid and oriented at the time of the interview, and attending the institution for at least 3 months.

**Table 1:** Number of participants interviewed by type of service. Vitória da Conquista, Brazil, 2019

	Health Professionals	Policy Makers	MH Users
**CAPS** (II, AD, and IA)			
2 nurses		3	3
3 psychologists			
2 social workers			
2 psychiatrists			
**Primary Care** (eSF and NASF)			
3 nurses		2	1
3 psychologists			
**Street Clinic**		–	–
1 social worker			
1 psychologist			
**General Hospital**			
1 nurse		2	1
1 psychologist			
1 social worker			
1 psychiatrist			
**Total**	21	7	5

Interviews were conducted using a script focusing on (supplementary file 1) focusing on (i) how users have access to MH services; (ii) how they are cared for in the services and their needs acknowledged; (iii) the existence of coordinated care mechanisms over time, including family involvement and social instruments; (iv) types of activities that are developed in the services for comprehensive care; (v) what approaches are taken to users in crisis and where. The script and the emphasis of the approach were adapted for each participant group. The average interview duration was 60 min.

Direct observation was used to identify the physical structure and organisational aspects of services, reception of users, types of treatment offered, possibility of continuity of treatment, articulation between RAPS services, and promotion of autonomy and articulation between services (supplementary file 1). The services observed were CAPS IA, CAPS II, CAPS AD III, 2 eSF, and MH beds in the general hospital. Direct observation was carried out between July and September 2019, with a total duration of 130 h.

### Analysis

We used an analysis framework, the development of which constituted a dynamic and interactive process of research in the international literature and integrated work between authors MLS and JPBJ. We consider comprehensive MH care a complex process that involves people from different social spaces, diverse health services, and intersectoral practices.

We used three steps to develop the analysis framework. First, we chose methodological sources that take a comprehensive and people-centred mental health approach [4, 6, 7, 25, 28, 29]. Second, we identified the main dimensions relating to comprehensive MH care, considering the axes involved in MH care development in primary health care, specialised services, and hospital care. We contemplated aspects of the services offered, how people access these services, and how care coordination and collaboration occur among professionals, users, and families. We also included factors beyond clinical care that involved intersectoral actions for the social inclusion of people with MH problems. Third, we developed an interactive process of systematisation and refinement of the study dimensions and the sub-dimensions that make up each dimension. We looked for differences, similarities, and intersections between the sub-dimensions and dimensions.

**Fig. 1:**
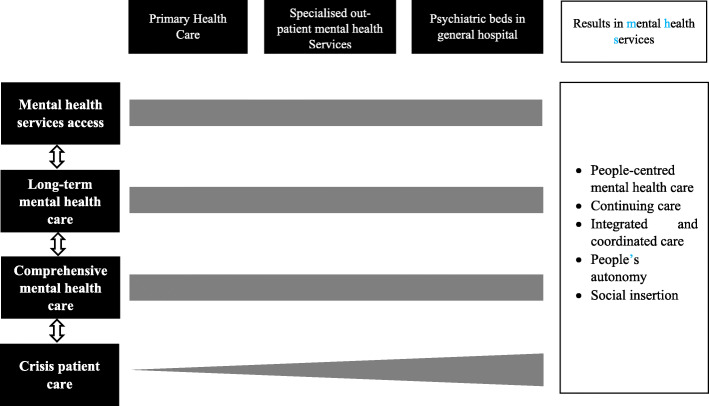
Dimensions of mental health care and health care levels. Legend- The figure illustrates the relationship between MH dimensions and health care levels. On the left side, bidirectional arrows illustrate the interdependence of the dimensions. Thus, the figure expresses the non-rigid delimitation between the elements of each dimension. There may be characteristics of sub-dimensions present in more than one dimension. The horizontal shaded bars express the equal responsibility among the three levels for ensuring service access, long-term MH care, and comprehensive MH care. In the dimension of crisis patient care, the wedge-shaped line indicates that although all three levels should provide care in acute situations, there is an increasing level of capacity to resolve these situations

The analysis framework developed thereby of four dimensions: (1) mental health services access, (2) long-term mental health care, (3) comprehensive mental health care, and (4) crisis patient care. The dimensions and sub-dimensions analysed are presented in Table 2. Although each dimension has specific characteristics, as shown below, we highlight that these components are intertwined and have characteristics in common. Comprehensive and effective MH care should be expressed by the interrelationship between and inseparability of the different dimensions and levels of service provision (Fig. 1).

**Table 2:** Analysis framework of mental health care performance

Dimension	Sub-dimensions	Sub-dimensions description
Mental Health access services	• Pathways to access mental health services	Entry mechanisms and/or ways adopted to obtain MH care.
	• Reception and triage	Welcoming of patients, qualified persons listen to descriptions of problems, assessment of the severity of the case, and definition of MH care needs.
	• Barriers to access	Gaps and impasses that make it difficult or impossible for patients to get treatment in MH services.
Long-term mental health care	• Deinstitutionalisation actions	Development of actions oriented towards promoting anti-asylum MH care, patient autonomy, and community insertion.
	• Coordinated and collaborative care	Integrated practices between services to ensure comprehensive MH care in the long term. This requires formalisation of communication and clear responsibilities definitions of professionals and services.
	• Shared goals and vision	Individualised care plan in MH with the establishment of common goals shared between professionals and users.
	• Family participation	Integration of the family in the patient’s therapeutic and recovery process, sharing responsibilities with the health teams. It also covers spaces for listening and MH care with these family members.
Comprehensive mental health care	• Out-patient/ambulatory clinics	Clinical and individualised consultations with health professionals, considering the patients’ unique needs.
	• Pharmacological treatments	Free provision of psychotropic drugs for patients in need of drug treatment by MH services.
	• Talking and psycho-social treatment	Interventions that value the dimension of subjectivity and listening. They include psychological or community support groups, therapeutic workshops, and individual psychological consultations.
	• Intersectoral interventions	Articulation with other social sector agencies with the aim of achieving comprehensive and sustainable plans rather than simple discussions of clinical issues. This involves social inclusion of patients, promotion of autonomy, and income generation.
Crisis patient care	• Crisis resolution teams	Multidisciplinary teams that carry out intensive and resolute treatment in an MH crisis, aimed at stabilizing the patient or avoid hospitalisation.
	• Therapeutic approach for the crisis patient	Types of intervention and therapeutic methods used to stabilise crisis patient.
	• Follow-up of crisis patient	Existence of mechanisms and protocols for continuity of care in the psychosocial care network after the patient’s acute crisis.
	• Difficulties to manage the crisis patient	Gaps and impasses that make it difficult or impossible to garner the assistance of patients in crisis.

Mental health services access consists of entry mechanisms and/or access routes adopted for care. It considers how users enter services: free demand, referrals from other services, or by court order. This dimension comprises access to MH services, reception and triage practices, and barriers to access.

Long-term mental health care is defined as establishing long-term relationships between health services and users, according to the needs of the subjects and family members, to achieve continued care. It assumes that long-term care should involve articulation between professionals and the various RAPS services, with shared objectives. Family involvement and deinstitutionalisation actions are also important elements that have been identified as necessary in Brazilian MH care policy [17, 30]. The following sub-dimensions were analysed: deinstitutionalisation actions, coordinated and collaborative care, shared goals and vision, and family participation.

Comprehensive mental health care entails the use of multiplicity of care technologies to achieve integrated care that responds to individual needs. This dimension concerns the scope of actions offered and the problem-solving capacity of the services, with emphasis on comprehensiveness of care. Hence, the following sub-dimensions were considered: outpatient or ambulatory clinics, pharmacological treatments, talking and psychosocial treatment, and intersectoral interventions.

Crisis patient care concerns the capacity of services to provide care for and resolve MH crisis situations. It aims to identify services that have crisis resolution teams, approaches for patients in symptom exacerbation, case follow-up, and existing limitations. The analysis involved the three levels of care – primary health care, specialised services, and hospital care – and comprised the following sub-dimensions: crisis resolution teams, therapeutic approach for crisis patients, follow-up of crisis patients, and difficulties in managing crisis patients.

Materials obtained in interviews and participant observation were transcribed, categorised, and codified using techniques proposed by Strauss and Corbin [31]. The categories were agglutinated using semantic content criteria.

### Ethics

The Research Ethics Committee of the Multidisciplinary Institute of Health of the Federal University of Bahia approved the study (opinion no. 3.374.680). All participants signed an informed consent form.

## Results

### Mental health services access

Access to MH services takes diverse forms, such as direct access, referrals from other services, active search of cases, or judicial decisions. Direct access was the main entry mechanism, although we observed variations between service types. Most services function as an open door for MH care, which facilitates self-referral by users. Self-referral services follow the logic of direct access for users. Thus, the person can directly seek MH care services without the referral need by another professional.

In the APS, direct access occurred through searching for medical consultation or participation in MH therapeutic groups. These groups were developed by eSF physicians and nurses or NASF professionals. The Street Clinic service is intended for homeless people and especially provides care for crack and other drug users. In this service, attendance ran on free demand and active search by users. In secondary care, in addition to self-seeking, access also occurred through referrals from the APS, the general hospital, or social support centres.

Hospital care mainly aimed at people in a psychiatric crisis. Psychomotor agitation, aggression, suicide risk, and drug withdrawal crises were defined as emergencies for hospital admission. In this case, entry occurred by SAMU, referrals from the APS and community MH services, and compulsory court-ordered hospitalisations.

*Most emergency room patients are from crisis services or* via *regulation. Some cases in which the family cannot find any solution come here as well.* (Interview 25: Policy maker)

All services had reception and triage mechanisms. Reception refers to as the moment of hearing the problems, symptomatological assessment, and situational comprehension of the case. After triage, it is decided whether the person will be treated in the service itself or forwarded to another service. It is emphasised that reception is broader than simple triage of cases. Reception considers that there is some degree of suffering that triggered the search for care, so the user should always be welcomed and oriented upon arrival.

*You have to go through a triage. I was attended by a nurse, social worker, and a team of doctors. So, I began to have care with this doctor who accompanies me to this day.* (Interview 23: User)

We identified several access barriers that constitute limitations of MH care. The main reason was insufficient availability of services. All care levels had insufficient vacancies for consultations, user waiting lists, insufficient doctors, or lack of beds for hospitalisation of severe cases.

*We have a population of almost 7000 inhabitants for one team. So how are we going to handle the demand?* (Interview 28: Professional)

*Some days, there’s only one doctor to take care of everything. Attending the complications of the ward, attending the emergency room, and attending the outpatient clinic. It’s heavy, it’s difficult.* (Interview 26: Professional)

*We have three vacancies for reception a day. One arrives and he’s got the vacancy, he’s already attended, right? However, there’s no way to meet all the demand.* (Interview 8: Professional)

An important contradiction was identified in reception and triage. The principle of reception is that all people who arrive at the service can be attended and heard. However, there were few reception vacancies in the CAPS. Some cases of family members and children spending the night at the door of the institution in an attempt to guarantee a place for attendance were reported.

### Long-term mental health care

The results of the variables showed that long-term MH care showed important advances in some respects, while in others, the practices revealed limitations in continued care.

We identified dichotomous relationships in deinstitutionalisation actions. Both specialised and hospital services acted to promote users’ autonomy and community insertion through territorial activities, articulation with social services, and actions to promote self-care and income generation. On the other hand, these services also maintained centralised care under the aegis of user protection. In some situations, APS services promoted localised actions, while in others they neglected care and referred clients to specialised services in cases that could be accompanied by eSF.

*I feel afraid to refer them back to primary care sometimes. I have a feeling if we refer, it won’t be taken care of. (*Interview 13: Policy maker)

Mobilisation of family members and legal sectors for compulsory and prolonged hospitalisations constituted obstacles to deinstitutionalisation. The non-existence of a therapeutic residence for de-hospitalisation of patients who had been hospitalised for years was also considered as a limiting factor.

Collaborative work is a challenge to be overcome for MH care. Although teamwork articulation initiatives were observed, frictions in communication, divergence in the forms of service provision, and hierarchisation of professions hindered execution of coordinated work.

*You have the task division, every professional in his place. There’s no exchange.* [...] *They don’t look at the individual as a whole.* (Interview 22: Professional)

The absence of clear definitions of the role of eSF and professional responsibilities in relation to users with psychiatric disorders made it difficult to perform coordinated work.

*Just knowing that someone is registered with CAPS, regardless of whether they are in crisis or not, leads some PHC professionals to not want to attend. They said it couldn’t be there, you know?* (Interview 12: Professional)

In the CAPS, team meetings and the presence of the case manager provided actions with greater integration and horizontalisation. We observed frequent distancing and isolation from the work of physicians to other professionals. In the hospital context, although there are meetings of the MH teams, fragmented practices and difficulties for collaborative work were in force.

Shared goals and visions between professionals and users were recognised as essential for development of continued care and, at the same time, verified as tools that are difficult to operate in the daily life of services.

*It doesn’t work together. For me, the Singular Therapeutic Project is the hardest thing to put into practice.* (Interview 25: Policy maker)

Treatment over time works better when the family itself is an ally in the therapeutic process and shares responsibilities with health teams. However, family relationships can also hinder continued and deinstitutionalised care. We identified situations in which families, because of socioeconomic difficulties, overload, or cultural aspects, resisted caring for family members with mental suffering at home and sought hospitalisation, often resorting to legal action.

*The family said, ‘We’re not taking him home’. [...] So the family went to the district attorney’s office so they could leave the patient hospitalised here.* (Interview 26: Professional)

In specialised services, follow-up and support actions were developed for families, with maintenance of meetings and talks with family members. In the other components of the RAPS, there was no systematised participation of family members.

### Comprehensive mental health care

The services studied provided individualised clinical care to users. We identified a great demand for clinical care in the APS and specialised care with long queues. In the APS, not all teams provide clinical care to cases of mental distress, which depends on the profile and sensitivity of professionals and teams. Given the difficulties of access to psychologists and psychiatrists, users resort to supplementary care in the private sector. Higher-income users in particular seek private MH care by paying out-of-pocket. Thus, there were situations of people being treated by SUS and at the same time making use of supplementary private services to obtain individualized clinical care.

Psychotropic drugs are provided in all services. However, we found a lack of basic medications, leading to restrictions on therapeutic options. Basic psychotropic drugs are those used in the most frequent MH problems and should always be available in the RAPS services; however, we have identified an underprovision of these psychotropic drugs. We observed the context of overvaluation of drug care.

*I get some medications through the SUS, others I buy. Because the SUS doesn’t provide all.* (Interview 23: User)

Talking and psychological treatment are widely used in the eSF and CAPS, although with consistent limitations in provision. Users had difficulties in accessing individual psychotherapy because of the high demand and low availability of professionals. Talking and psychological treatment are carried out mostly through therapeutic groups. This method was justified as a strategy to support more people and enable socialisation, bond building, and development of cognitive skills.

*It started to get too long for individual care, to have waiting lists, so let’s do group care*. (Interview 9: Policy maker)

*I’m leaving with the psychiatrist scheduled. No psychologist. There’s a line, so you have to wait. The only one that is harder to get is individual psychotherapy.* (Interview 11: Professional)

Acceptance and adherence to group therapy varied among services. Given the situation of low group membership and strong valuation of pharmacological treatment, some services reconciled the two elements by creating groups associated with clinical evaluation and dispensing medications. However, in some services, collective activities assumed the mere function of changing prescriptions for the dispensal of controlled drugs.

*The mental health group is the prescription exchange group. It is not just a person with a mental disorder who goes to this group. Many family members will collect prescriptions for relatives; the user is not even there!* (Interview 31: Professional)

The services developed intersectoral interventions to reduce stigma and violence and foster artistic expression and work inclusion. Although several initiatives were reported, such as inclusion in educational actions, artistic and literary production, and actions to promote well-being, professionals and users highlighted the need to expand this type of activity to overcome prevailing traditional biomedical practices and stigma.

### Crisis patient care

The RAPS demonstrated serious difficulties in crisis patient care and generally had limited management capacity for these conditions. We found considerable difficulties for patients receiving care in crisis situations.

All CAPS and the general hospital maintained crisis resolution teams, although with heterogeneous resolution capacities. CAPS III, being a service for alcohol and other drug users, only attended crises involving this target audience. CAPS II and CAPS IA had limitations in crisis management because of limited infrastructure and excess demand. We identified hospital overload and lack of vacancies in psychiatric beds. The difficulties of the other units in attending cases with symptomatological exacerbation were reported as the main factor in hospital overload.

*We do not have a dedicated space for emergency or for a teenager or child with mental disorder and in crisis. In the end, we improvise what is possible, right?* (Interview 10: Professional)

*It compensates for the discharge. For every patient who leaves, we have three more addicts out there that can enter.* (Interview 26: Professional)

In the hospital, crisis management occurred predominantly through physical and drug restraints, with other care strategies being underdeveloped. Attempts to reduce overmedication practices were also observed. Although it is a 24-h hospital, there were difficulties in attending users on weekends and outside business hours. This reality has exacerbated violent measures aimed at people in crisis, such as home chains and violent restraints in other services.

*There is a room where you place the person, if necessary, contained physically or chemically to be able to check in.* (Interview 20: Professional)

*If the crisis occurs on Friday, the patient is tied up from Friday to Monday, until he can come here.* (Interview 26: Professional)

Follow-up of crisis patients was not observed in the services assessed. The difficulties in follow-up of cases were influenced by lack of care flow.

*There is no protocol after discharge. [...] Some get lost, that’s the truth. They don’t return.* (Interview 31: Professional)

*We don’t get so involved in this moment of thinking after leaving the hospital. So, I don’t know how this follow-up would actually be.* (Interview 20: Professional)

We found no evidence of formal and systematised communication mechanisms between hospitals and other units that provide MH care.

## Discussion

This study’s findings demonstrate the presence of innovative practices in the provision of MH care in Brazil. We also identified difficulties that hinder the provision of comprehensive MH care. These factors can be classified as facilitators and barriers. Considering the four dimensions of the analysis framework, the main facilitators were implemented localised care strategies and support with an expanded view of MH; open door policies; street clinic teams; reception and triage; the development of deinstitutionalisation actions; family involvement; the availability of multiple care technologies; and intersectoral actions. The most common barriers we found were limited access to various services; restricted reception; lack of trust between professionals and limitations in collaborative care; overvaluation of psychotherapeutic drugs; and limitations in the acute care and use of violent practices.

The examination of facilitators and barriers allows us to identify the factors that belong to the same dimensions or sub-dimensions. Thus, they act simultaneously as both promoters and threats to comprehensive MH care due to the advances and restrictions in the Mental Health Policy in Brazil. With RPB, the country achieved important advances in MH legislation and instituted a person-centred, community-based care model. However, structural, economic, and contextual difficulties restrict the scope of some of the implemented changes, although the extent of such changes was intended to be greater. The discussion of the four dimensions of our findings highlights key aspects of this context.

Broad and quality mental health services access is considered essential for the overall response of health systems to MH problems [6]. Therefore, MH care should be included as an essential component of universal health coverage to ensure quality access to all people with mental problems. In Brazil, access to health services is guaranteed by the SUS. Community MH services have an ‘open door’ policy, considered strategic to consolidate transition from hospital to the community [19]. However, there are substantial difficulties in ensuring MH care to all people who seek the RAPS. Our study demonstrated that access is still limited due to long waiting lists, and access varies between institutions.

The main access barrier corresponds to the inability of services to meet the high demand for MH care. Results showed that users who need and seek care are not always assisted in a timely manner. The limited supply situation was aggravated by organisational barriers including inflexible protocols and practices and lack of systematised care flows between services. Barriers to accessing MH services are an important limitation and challenge to be overcome in LMICs, as in African [28, 32], Asian [7], and Latin American countries [13].

A prominent finding was the existence and use of reception mechanisms, especially in specialised care, which enables transitioning from a solely symptomatic perspective toward a comprehensive approach to users. In Brazil, reception (in Portuguese, *acolhimento*) is a guideline of the National Humanisation Policy. Reception is defined as a technology of relationships between professionals and patient for qualified listening and to facilitate bond establishment and ensure timely access for all people seeking services [33]. These actions aim to expand SUS resolutivity and health services’ responsibility to patients [34]. However, our finding showed that service overload distorted the concept of reception, which is based on welcoming everyone who seeks psychosocial support. Thus, CAPS frequently began allowing small numbers of daily reception vacancies.

Another important initiative to expand care in MH was creating street clinic teams. These teams allowed greater access to people without fixed housing who were dependent on psychoactive substances or had some type of mental disorder. We consider street clinics an innovative strategy for inclusion of extremely vulnerable populations with a history of invisibility in ensuring social protection.

Street clinic teams are multi-professional teams formed by nurses, psychologists, social workers, and mid-level professionals with the aim of providing comprehensive health care to the people in conditions of greatest vulnerability. The actions developed are integrated with PHC and, when necessary, with CAPS and emergency services. An experience reported by Horspool et al. [35] demonstrated that street triage services helped to compensate for the lack of crisis teams. In Brazil, expanding street clinic teams have potential to be an important way to fight against crack and other drugs and reduce the demand for specialised and emergency services.

Our results indicate that deinstitutionalisation actions enhanced development of long-term mental health care. We identified actions to promote autonomy and community insertion in people with mental disorders. Brazil’s MH policy presupposes deinstitutionalising care in an open environment, valuing autonomy and social participation [19]. These assumptions are consistent with people-centred MH services [36] and MH recovery [37] as well as World Health Organisation [5] recommendations for providing care with a participatory and emancipatory focus.

However, the coexistence of traditional and paternalistic biomedical practices in community services relegates users to passivity and the need for guardianship, reproduces the logic which it proposes to overcome, and hinders leaving specialised outpatient MH care. Leaving specialised services can be understood as a process of building full autonomy, so that users have an independent life and do not always need to be supervised by community MH services. Deinstitutionalisation actions should also be an objective of services of open environment. Our results demonstrated a movement of re-institutionalisation in the community, as reported by Killaspy [38]. Gurung et al. [39] and Hall et al. [4] also evidenced similar situations in open systems.

Barriers to deinstitutionalisation were associated with the weaknesses of integrated and coordinated care. Unclear roles and lack of trust between the services hindered both deinstitutionalisation actions and integration between MH services and primary health centres.

In coordinated and collaborative care, team interventions are indispensable to achieve the objectives of integrated and longitudinal care because they allow redesign of care delivery and recognition of users’ complex needs [40]. In our study, multidisciplinary meetings and the presence of the case manager in the CAPS favoured team communication and patient follow-up. However, the results also demonstrated the need to strengthen communication strategies between professionals and services and overcome fragmentation of practices and rigid work divisions.

In contrast to our findings, Ramanuj et al. [41] identified the presence of collaborative care, clear roles and responsibilities, care coordination, and effective communication between the behavioural health and PHC services in New York. The authors conclude that these factors act as facilitators for integrated care provision. However, they also highlighted that these factors can rarely facilitate integration in isolation.

Family participation was identified as essential for long-term care and good social recovery. We highlight the difficulties of some families in providing adequate support to people with mental disorders. Our study identified situations of compulsory hospitalisation sought by judicial means and weaknesses of services in monitoring and supporting families. In these situations, court-ordered hospitalisations can relieve caregivers [4]. Liang et al. [7] highlighted the inclusion of family members as an indispensable element of MH care both for potentiating longitudinal care and for broadening approaches to the impacts of human suffering.

The manicomial model of permanent hospitalisation that long existed in Brazil affected familial relationships by breaking affective bonds and reducing people’s sense of responsibility for family members’ health care. With the advent of the community approach, there have been advances in the re-signification of family relationships and responsibility of relatives. Although the findings show that family participation is a facilitator of MH care, this was present only in CAPS. We emphasise that achieving comprehensive MH care also requires family involvement in other services, notably PHC and hospital care. Effective long-term therapeutic plans require support from and for families in all components of the RAPS.

As for comprehensive mental health care*,* we identified opportunities in the services using multiple care technologies. The results showed the development of artistic activities and community actions in the APS and CAPS that transcend the solely symptomatological approach and are close to comprehensive MH care. In turn, we also identified structural barriers such as lack of medications, insufficient supply of talking and psychological treatment, and limitations in intersectoral interventions. These weaknesses affect the quality of care offered, contribute to worsening of cases, and lead to exclusion of users who cannot afford medications or treatments in the private sector.

Despite the wide range of actions developed in the services indicating a direction for the comprehensive MH approach, structural barriers and cultural factors limit this broader perspective. Therapeutic groups are an emblematic example of this situation. Our findings highlight that due to lack of workforce, many therapeutic groups have been created to compensate for the limited provision of individual psychotherapy. In this context, existing barriers have prevented the achievement of the objectives of health promotion, subjectivity valorisation, and the exchange of experiences between patients. Thus, many groups have been transformed largely into spaces for dispensing medicines, strengthening the reproduction of the biomedical model in the community.

Crisis patient care requires coordinated articulation between multiple service platforms to identify situations and promote rapid and resolutive responses [5]. International efforts tend to value quality care in psychiatric wards in general hospitals in place of psychiatric hospitals [5, 6, 42], and ensure that extra hospital services can contribute to management of crisis conditions and reduce hospitalisation [43, 44]. Our study revealed that the RAPS had a great weakness in attending to users in crisis. RAPS services lack adequate structure and sufficient professionals to attend emergencies. Such weaknesses enhanced situations of lack of assistance, practices of violence, and worsening of suffering by users and family members.

Current guidelines underlying global MH advocate ensuring the dignity of the person in mental distress, in line with Human Rights Conventions [29]. However, the reality studied by us in Brazil revealed shackling situations, inadequate restraints*,* and private prisons – unacceptable conditions for the present day. Historical attributions of dangerousness, threat, and non-citizenship to people with mental suffering [32] still sustain and validate the reproduction of violent and inhumane practices. Given this study’s findings, we emphasise the need to develop changes in the care model from psychiatric hospitals to the community environment through integrated services, with care support capable of meeting all people’s needs and moving more effectively towards dismantling of still prevalent stigmas.

The results of our study confirm analyses [15, 19, 20] showing that the experience of MH care in Brazil has made important advances towards comprehensive MH care. However, our research also showed that there are still inadequacies and weaknesses in the care developed by the RAPS. Although the SUS has universal and comprehensive care as principles, Brazil still has difficulties in ensuring the right to health, especially because of high existing social inequality and low amounts of resources allocated to health services [14, 45]. Strict fiscal austerity policies implemented in 2016 undermined the sustainability of the SUS [46], a situation aggravated by President Bolsonaro’s government, which aims to implement an ultraliberal agenda and dismantle public policies. This situation tends to weaken community MH and exacerbates obstacles identified in our study.

The country’s current political and economic context threatens the advances achieved by community-based MH policy and guided by psychosocial care. The approval of the Constitutional Amendment 29, in 2016, limited federal primary expenditure on health over the next 20 years [45], with great impact on the already restricted funding of the SUS and on the RAPS budget. Thus, the restriction in the availability of some services and the limitations in hiring health professionals demonstrated in this research, tend to be further hampered.

In addition to the general weakening of SUS, recent changes in the National Mental Health Policy represent a move back to the asylum model. Actions such as the inclusion of the psychiatric hospital in the RAPS, the public funding to religious therapeutic communities that operate with practices of restriction of freedom and segregation of people in mental suffering, and the opening of psychiatric hospitals for the admission of children and adolescents [22] go against RPB ideas and threaten social recovery and people-centred care strategies. Moreover, these aspects reinforce the stigma of the person with mental problems as someone who needs to be excluded from living in society. According to Onocko-Campos [23], the current public administration in Brazil does not adhere to scientific evidence and disrespects civil, political and social rights. Thus, the innovative actions revealed in our study may not continue if the policies advance further that take an asylums approach – fragmented, strictly clinical and rights-limiting.

### Strengths and limitations

A strength of the study was the wide range of stakeholders interviewed. We interviewed professionals from many RAPS services, policy makers, managers of specialized services and hospitals, and users. Another strength was the extensive observation period of the services.

Our study has limitations. All participants were advocates of the RPB and the community MH care model, so there may have been social desirability bias. We attempted to overcome this limitation by devoting special attention to field observation data to identify the dynamics of service function and the relationships established between professionals and users. Despite the wide range of stakeholders, the study had a small number of users interviewed. We had difficulties in selecting regular service users who were lucid and oriented during the data collection period. Another limitation concerns the non-inclusion of mobile emergency medical services in the study.

## Conclusion

Our study revealed that the RAPS studied showed advances towards comprehensive MH care, such as community care, use of multiple care technologies, teams with case managers, and actions to promote autonomy and community integration. However, we also found limitations in the care provided, forms of exclusion, and violent practices, which are characteristics still present in LMICs. The advances and limitations we find suggest that conflicting theoretical and practical conceptions of care models coexist in parallel in the MH area. We highlight the need to expand access and integration between the various service platforms, foster support to families, strengthen unique care strategies aimed at the territory, promote user autonomy, and systematise management actions to crisis patients that provide dignified, comprehensive, and continuous care. MH care is inseparable from the healthcare model in force in the country. Thus, given current attacks on the SUS, defending Brazilian Psychiatric Reform is crucial to defending a universal, equitable, and integrated Brazilian health system.

## Supplementary Information

**Additional file 1.**

## Data Availability

Data can be requested from the corresponding author.

## Abbreviations

MH: Mental Health
CAPS: Psychosocial Care Centres (*Centros de Atenção Psicossocial*)
CAPS-II: CAPS for severe and persistent mental disorders
CAPS-AD: CAPS for users of alcohol and other drugs
CAPS-IA: CAPS intended for children and adolescents
eSF: Family Health Teams (*Equipes de Saúde da Família* – eSF)
NASF: Family Health Support Centres (*Núcleos Ampliados de Saúde da Família*)
RAPS: Psychosocial Care Network (*Rede de Atenção Psicossocial*)
RPB: Brazilian Psychiatric Reform (*Reforma Psiquiátrica Brasileira*)
SUS: Unified Health System (*Sistema Único de Saúde*)
