# Supplementary material for: Towards comprehensive mental health care: experiences and challenges of psychosocial care in Brazil
Source: BMC Public Health. 2021 Jul 8;21:1352. doi: 10.1186/s12889-021-11397-1 (PMC8268580; doi:10.1186/s12889-021-11397-1)
Supplement: Supplementary file 1 — Additional file 1. [file 12889_2021_11397_MOESM1_ESM.docx]

**Towards comprehensive mental health care: experiences and challenges of psychosocial care in Brazil**

**Authors**

Mariá Lanzotti Sampaio. Multidisciplinary Institute of Health, Federal University of Bahia (UFBA), Vitória da Conquista, Brazil. Psycologist and Master in Collective Health.

José Patrício Bispo Júnior. Multidisciplinary Institute of Health, Federal University of Bahia (UFBA), Vitória da Conquista, Brazil. Doctor in Public Health.

**Correspondence:** José Patrício Bispo Júnior. Multidisciplinary Institute of Health, Federal University of Bahia (UFBA). Rua Hormindo Barros, 58. Bairro Candeias. Vitória da Conquista, Bahia, Brasil. E-mail: [jpatricio@ufba.br](mailto:jpatricio@ufba.br)

**Supplementary data**

**Interview guide** – **Health Managers and Health Professionals**

1. Can you tell me a little about your role in Psychosocial Care Network?

2. For you, what is the purpose of mental health care in this institution?

3. What do you think about mental health users and/or about person that use alcohol and other drugs?

4. What do you think about the deinstitutionalization process?

5. Are there legal or normative documents that guide mental health care at this institution? Can you tell me about them?

6. How does the user access the service?

7. How does scheduling occur for health professionals? (Is there reception and triage?)

8. You can tell me about the activities developed at this institution. (Which professionals participate?)

9. For you, how this service contributes to the deinstitutionalization process?

10. What are the clinical services offered here?

11. Is there long-term mental health care for users with clinical demands?

12. How does the family participate in the care of mental health users?

13. Are there user participation actions or mechanisms for giving an opinion on the activities offered? Tell me a little about that.

14. How is collaborative work developed in this institution?

15. How is this institution articulating the work with the other services of the Psychosocial Care Network?

16. Would you like to say something more about mental health care?

**Interview guide** – **Mental health Users**

1. What do you think about mental health?

2. What do you consider good mental health care?

3. Have you heard of or attended a psychiatric hospital? What do you think of this institution?

4. Have you ever received care in a psychiatric hospital? What do you think of this institution?

5. Have you ever participated or given an opinion on mental health care offered? Tell me a little bit about it.

6. Can you tell me how it was the first time that you were assisted in this service? (Why did you come? How did you hear about the service? How was your first contact?).

7. Can you tell me a little bit about what mental health activities you participate in at this institution? Where do they occur? Which professionals participate?

8. Have you helped in the elaboration or in the choice of the theme of any activity?

9. Do you participate in other actions, such as cultural actions and exhibitions? How do these activities happen?

10. How do you evaluate the mental health care offered to you?

11. How does your family participate in your treatment?

12. What do you think can improve the mental health care that is offered for you?

13. Have you been referred to another service? How was it? Can you tell me an example?

14. Do you receive mental health care at PHC centers? Can you tell me about it?

15. Have you ever used emergency mental health services? Which service did you use? How was it?

16. Would you like to say something more about mental health care?

**Direct Observation Guide**

| **Date:**  **Local / Health Service:**  **Observation time: to** |
| --- |
| **Physical structure**  (Existence of meeting rooms, consulting rooms and open spaces; chairs and ambience; and conservation status.) |
| **People and health professionals**  (Professionals involved in activities; professional-user and professional-professional relationship; articulation with other mental health services and articulation with social services) |
| **Types of mental health care that are developed** |
| **Other notes** |
